# P-637. Infection Incidence and Trajectory of Immune Recovery in Children, Adolescents, and Young Adults After Chemotherapy for Acute Lymphoblastic Leukemia

**DOI:** 10.1093/ofid/ofae631.834

**Published:** 2025-01-29

**Authors:** Morgan Zalot, Leena Chehab, Jenna Rossoff, David Hoogstra, Subhi Karthikeyan, Monica Newmark, Ryleigh VanDuine, Mary Beth Readwin, Laura A Vella, Brian T Fisher

**Affiliations:** Children's Hospital of Philadelphia, Philadelphia, Pennsylvania; Children's Hospital of Philadelphia, Philadelphia, Pennsylvania; Ann & Robert H. Lurie Children's Hospital of Chicago, Chicago, Illinois; Helen DeVos Children's Hospital at Corewell Health, Grand Rapids, Michigan; Childrens Hospital of Philadelphia, Philadelphia, Pennsylvania; Ann & Robert H. Lurie Children's Hospital of Chicago, Chicago, Illinois; Corewell Health, Helen DeVos Children's Hospital, GRAND RAPIDS, Michigan; Corewell Health West, Grand Rapids, Michigan; Children's Hospital of Philadelphia, University of Pennsylvania, Philadelphia, Pennsylvania; Children’s Hospital of Philadelphia, Philadelphia, Pennsylvania

## Abstract

**Background:**

Advances in chemotherapy for acute lymphoblastic leukemia (ALL) have improved survival rates, but survivors remain vulnerable to infection after completing chemotherapy. Data have shown increased infection risk for pediatric ALL survivors compared to healthy children, yet few studies have prospectively tracked infection rates alongside immune recovery. This study aims to quantify infection incidence and describe the trajectory of immune reconstitution in the year after ALL therapy completion.

**Figure:**
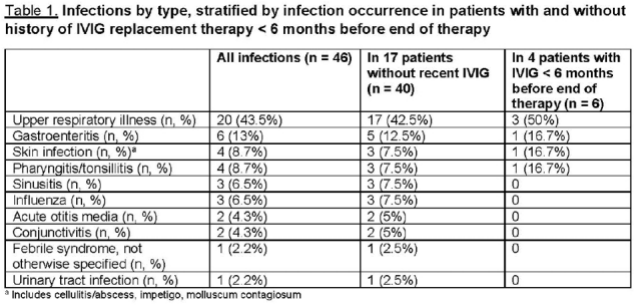

**Methods:**

Patients aged 3-30 completing ALL therapy at a pediatric center are being followed for the first year off-therapy. Total immunoglobulin-G (IgG) and antibody (Ab)-specific IgG levels for varicella, measles, and 23 pneumococcal serotypes are measured at 3, 6, and 12 months. Infection events are recorded monthly to calculate infection incidence per 1000 patient-days.

**Figure:**
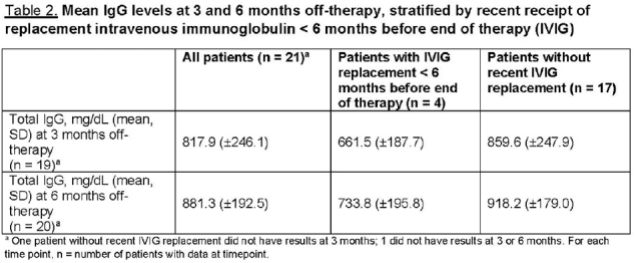

**Results:**

To date, 21 patients have been followed for ≥ 6 months. Median age at ALL diagnosis was 7.3 years (IQR: 3.4-10.1). 67% of patients had ≥ 1 infections; 93% of those with infections had ≥ 2. The overall infection incidence rate was 6.1 per 1000 patient-days. Of 46 infections, upper respiratory illness (20, 43.5%) and gastroenteritis (6, 13%) were most common (Table 1). 17 of 21 patients had not received intravenous immunoglobulin replacement (IVIG) within 6 months of therapy completion; their mean total IgG was 859.6 mg/dL at 3 months and 918.2 mg/dL at 6 months (Table 2). All 17 received ≥ 1 pneumococcus, varicella, and/or measles vaccine before ALL diagnosis. None had evidence of pneumococcus or varicella Ab recovery at 6 months, regardless of pre-ALL partial or full vaccination status (Tables 2-3). 7/11 patients fully vaccinated and 3/6 partially vaccinated for measles before diagnosis had normal Ab indices at 6 months off-therapy (Table 4).

**Figure:**
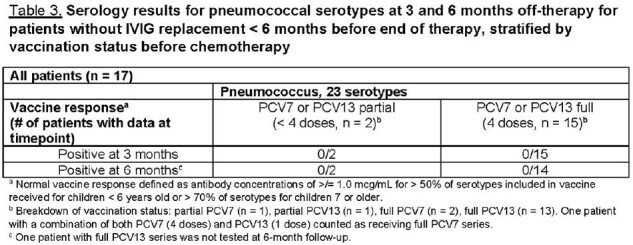

**Conclusion:**

Infections were common during the first year off-therapy, with many experiencing multiple illnesses. Total IgG levels were reasonable at 3 and 6 months, but there was no recovery of varicella or pneumococcus Ab, and only a subset of patients retained measles Ab. Continued assessment until 12-month follow-up for all 85 enrolled patients is necessary but preliminary data suggest potential need for Ab-informed revaccination.

**Figure:**
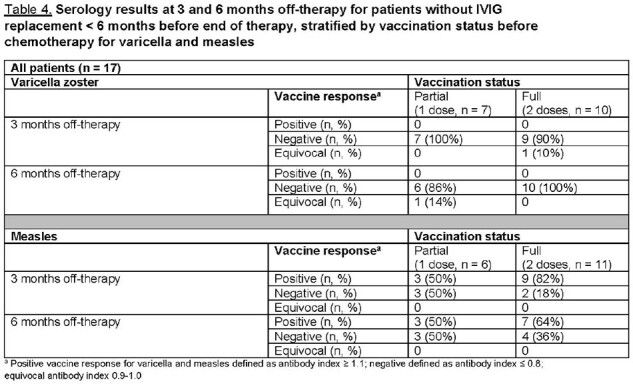

**Disclosures:**

**Brian T. Fisher, DO, MPH/MSCE**, Allovir: Grant/Research Support|Astellas: Data Safety Monitoring Board|Merck: Grant/Research Support|Pfizer: Grant/Research Support

